# A comparative study of *Toxoplasma gondii* seroprevalence in three healthy Chinese populations detected using native and recombinant antigens

**DOI:** 10.1186/1756-3305-6-241

**Published:** 2013-08-20

**Authors:** Xiaojing Sun, Huijun Lu, Boyin Jia, Zhiguang Chang, Shuai Peng, Jigang Yin, Qijun Chen, Ning Jiang

**Affiliations:** 1 Key Laboratory of Zoonosis, Ministry of Education, Jilin University, Xi’an Da Lu 5333, Changchun 5333, China

**Keywords:** *Toxoplasma gondii*, Prevalence, Antigens, China

## Abstract

**Background:**

Toxoplasmosis is one of the most common parasitic zoonoses. The seroprevalence of *Toxoplasma gondii* infection in humans varies widely worldwide. Detection of *Toxoplasma*-specific antibodies has been a gold standard method for both epidemiological investigation and clinical diagnosis. Genetic investigation indicated that there is a wide distribution of different genome types or variants of the parasite prevalent in different areas. Thus the reliability of using antigens from parasites of a single genome type for diagnosis and epidemiology purposes needs to be extensively evaluated.

**Methods:**

In this study, the prevalence of *T. gondii* infection among 880 clinically healthy individuals in China was systematically tested using crude soluble native antigens and purified recombinant antigens of type I and II *T. gondii*. The *T. gondii*-specific IgG and IgM in the sera was further confirmed using commercial Toxoplasmosis Diagnosis Kits and Western blot assays.

**Results:**

The sero-prevalence of *T. gondii*-specific IgG detected with crude native Type I and type II antigens was 12.2% and 11.3% respectively. Whereas the overall prevalence was more than 20% when combined with the results obtained with recombinant tachyzoite and bradyzoite antigens. There was an obvious variation in immune-recognition of parasite antigens among the individuals studied.

**Conclusions:**

The general prevalence of anti-*T. gondii* IgG in the study population was likely much higher than previously reported. The data also suggested that there is more genetic diversity among the *T. gondii* isolates in China. Further, combination of recombinant antigens with clear immuno-recognition will be able to generate more sensitive diagnostic results than those obtained with crude antigens of *T. gondii* tachyzoites.

## Background

*Toxoplasma gondii* is an intracellular parasite that can infect domestic, wild, and companion animals, and it also commonly infects humans [1]. The importance of this parasite in food safety, human health and animal husbandry has been well recognized. Though *T. gondii* infection in humans with a normal immune competence is asymptomatic in most cases, the parasites do pose threats to individuals who are immunocompromised, such as HIV carriers [2]. It has been estimated that up to one third of the world’s population has been infected by *T. gondii* with an endemicity from around 10% to 70% [1,3-5] and the prevalence is higher in warm and humid areas [6-8]. In several studies, patients with schizophrenia were found to have a higher tendency of *T. gondii* infection [9-12], but there has been no conclusive correlation between *T. gondii* infection and psychiatric disease [13].

*Toxoplasma gondii* displays significant genetic diversity in different geographical regions [14-16]. Currently, toxoplasmosis is diagnosed primarily by demonstrating parasite-specific IgM or IgG antibodies in serum samples. Most of the commercially available tests use *T. gondii* native antigens derived from the fast growing tachyzoites which may result in variations in accuracy of detection. Recombinant antigens have been suggested as diagnostic reagents but their reliability may need extensive experimental validation [17,18]. Further, *T. gondii* remains dormant as bradyzoites in immune competent individuals, which can convert to tachyzoites when the host immune defense system is compromised, and tachyzoites and bradyzoites do display different antigenic profiles [19]. Thus it is critical to select accurate antigens for diagnostic and epidemiological purposes.

In this study, we investigated the level of anti-*T. gondii* IgG and IgM in the sera of more than 800 Chinese individuals living in the southern and northern regions of China, comparing crude antigens of RH (Type I) and ME49 (Type II) strains and 12 recombinant antigens of either Type I or Type II *T. gondii*. The purpose is to compare the sensitivity and consistency in detection of *T. gondii* specific antibodies in the same set of samples with different antigens.

## Methods

### Study populations and serum samples

880 serum samples from clinically healthy individuals were collected in Changchun, Daqing and Shanghai areas in China from July 2006 to June 2012 as described previously [20]. The sera were collected with the consent of the volunteers. The study was carried out with permission from the Ethical Committee of Institute of Zoonosis, Jilin University, China.

### Antigens

Soluble native parasite antigens: *T. gondii* tachyzoites of RH and ME49 strains were cultivated in BHK (baby hamster kidney) cell lines as described earlier [21]. Briefly parasites released from host cells were harvested, washed in PBS and lysed by sonication. The insoluble component such as cell debris was eliminated by centrifugation (12,000 rpm for 30 min) and the soluble proteins, respectively termed RH-Ag and ME49-Ag were collected and diluted to a final concentration of 1 mg/ml in PBS for the serological test.

Recombinant antigens: To generate the recombinant antigens, the coding sequences of *T. gondii*, which including tachyzoite-specific (SAG1, SAG5A and SAG5D), bradyzoite-specific (BAG1, BSR4 and SRS9) and common antigens (MIC6, SRS4, GRA5, SUS1, SAG3, and SRS8) likely expressed in both tachyzoites and bradyzoites were amplified by the Polymerase Chain Reaction (PCR). Their accession numbers and primer sequences are listed in Table 1. Genomic DNA was isolated from the tachyzoites of the *T. gondii* RH and ME49 strains and was used as the template for PCR amplification of the coding sequences. The PCR reaction was carried out in a 25 μl reaction mixture containing 10 μM of each primer, 2.5 mM of each dNTP, 1.25 U of Ex Taq DNA polymerase (TAKARA), 0.5 μg DNA template, and 1 × Ex Taq buffer. The touchdown PCR was performed on the PCR System (Applied Biosystems, CA, USA) with a program of initial denaturation for 4 min at 94°C; 35 cycles of 94°C for 45 s, annealing for 45 s (initial temperature 61°C, then decreasing by 0.3°C/cycle), and 72°C for 2 min; and a final extension at 72°C for 10 min. The PCR products were respectively cloned into the plasmid pGEX-4T-1 (GE Healthsystems, Uppsala, Sweden) and PET-28a (Qiagen, Düsseldorf, Germany) to construct recombinant plasmids, which were subsequently confirmed by sequencing. The plasmids with correct sequences were transformed into BL21 competent cells and the His-tag and GST-tag fusion proteins were expressed and purified according to a standard protocol as described [22]. They were respectively named as RH-rAg, ME49-rAg and RH/ME49-rAg. The recombinant proteins were diluted to a final concentration of 1 mg/ml in PBS for the serological test.

**Table 1:** Primer sequences used in the Polymerase Chain Reaction (PCR)

**Gene name**	**NCBI accession no.**	**Primer sequence (5’-3’)**	**Amplified product (bp)**
SAG5D	AY190528.1	F-GATGCGACCGGAACGACCC	981
		R-ATATGTGCCAAGAAAGACA	
SAG5A	AF013968.3	F-GAGGTAACGAATGTTCTG	1002
		R-GTATGCACCGAAAGAAACA	
MIC6	AF110270.1	F-TCCCCGTTTTTTGCCTTTC	792
		R-ATGTCCACTTCCTTCCTC	
SRS4	XM_002366109.1	F-CCACATGAAGGTAGTTTG	1068
		R-GTAGGAACCAGCAAGAAGC	
GRA5	EU918733.1	F-TCAACGCGTGACGTAGGG	132
		R-TCCAGTCACCGCTGCTC	
SAG1	AY217784.1	F-TCGGATCCCCCTCTTGTT	867
		R-CGCGACACAAGCTGCGAT	
SUS1	U77677.1	F-TCTCCTCTTTCGATGGCC	1131
		R-GACGGCACCAAACATAGC	
SAG3	JF312642.1	F-GAGCACGGACTGTTCGTCG	1035
		R-GGCAGCCACATGCACAAGG	
BAG1	XM_002365075.1	F-ATGGCGCCGTCAGCATCGC	687
		R-CTTCACGCTGATTTGTTGCT	
BSR4	XM_002369844.1	F-GATAATCTTCTTGAAGGAC	1038
		R-GGCGGCCGCGCTAGAGGC	
SRS8	XM_002371596.1	F-GAACAACTCGGCGAAGG	1077
		R-AAGGGAACCGGCAAGAAGC	
SRS9	AF465609.2	F-GACAGTCTGCATGAAGGA	1077
		R-CAATGAAGCAACAACGAAC	

### Serological assay

Indirect ELISA assays were performed to measure the anti-*T. gondii* IgG level according to the standard protocol [23]. All reaction steps except coating and washing were performed at 37°C. Briefly, Maxisor micro-ELISA plates (Nalge Nunc International, IL, USA) were coated with 50 μl per well of the *T. gondii* antigens (RH-Ag, ME49-Ag and 12 recombinant antigens, respectively) in a concentration of 5 μg/ml at 4°C overnight. The plates were washed five times with PBS containing 0.05% Tween 20 and blocked for 3–4 h at 37°C with 100 μl per well of 0.5% BSA in PBS. After washing, 50 μl of each serum sample, diluted at 1:50 was added to the well in triplicates for 1 h. Alkaline phosphatase-labelled goat antihuman IgG (Sigma, St. Louis, USA, 1:2000 dilution) was added to the well after washing. Finally, 50 μl of NPP [4-Nitrophenyl phosphate disodium salt hexahydrate] (Sigma, St. Louis, USA) and 9.7% diethanolamine (pH 9.8) were used to detect antigen-antibody reactions. The plates were finally read in a Biotek 93 micro-ELISA auto-reader 808 at 405 nm. A human serum sample, which was previously confirmed with negative reactivity to *T. gondii* by the direct agglutination test was included as a negative control in every plate. The GST protein was used as control for the GST-tag fusion antigens. The cut-off point of OD value for a positive sample was set to be at least two times higher than that of the negative sample at any dilution point as described previously [20].

To further confirm the ELISA data, all positive and 50 randomly selected negative sera were further tested by the commercial *T. gondii* IgG/IgM Kits (Haitai Biological Pharmaceuticals Co., Ltd, China) that can respectively detect human IgG and IgM, and Western blot assays using soluble extract of *T. gondii*. The procedure of the commercial IgG/IgM kit was performed according to the manufacturer’s instruction [24]. For the purpose of Western blot assays, the parasite-derived soluble proteins were separated on a 12% SDS-PAGE gel and transferred onto nitrocellulose membranes (Bio-Rad, CA, USA). The membrane was cut into strips and incubated with the positive and negative sera (at 1:50 dilution) identified in the ELISA assays. Meanwhile, a serum of an individual previously confirmed with *T. gondii* infection was used as a positive control. The membrane strips were further incubated with an alkaline phosphatase-conjugated goat anti-human IgG antibody (1:20000 dilutions) after washing in TBST buffer (10 mM Tris, 150 mM NaCl, pH 8.0 and 0.05% Tween 20). Eventually, the strips were incubated with BCIP/NBT substrate solution to visualize the protein bands that were recognized by the specific antibodies.

### Statistical analysis

The results were statistically analyzed using the SPSS 18.0 software package. Chi-square test was used to analyze the anti-*T. gondii* IgG seroprevalence by using twelve *T. gondii* antigens. The differences were considered to be statistically significant when the *p* value was less than 0.05 [20].

## Results and discussion

**Figure 1:**
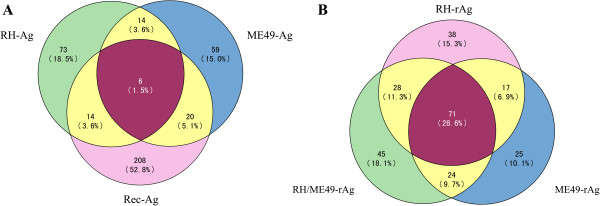
**Number of positive sera in a clinically healthy population detected with three kinds of antigen. A** shows the numbers and percentage (in bracket) of 394 sera with *T. gondii*-specific IgG detected by the crude antigens of RH, ME49 strain and recombinant antigens. **B** shows the numbers and percentage (in bracket) of the 208 positive sera with *T. gondii*-specific IgG detected by the recombinant antigens (RH-rAg, ME49-rAg, RH/ME49-rAg).

In this study, the prevalence of anti-*T. gondii* antibodies (primarily IgG) in 880 individuals was detected using tachyzoite-derived crude parasite antigens of RH (RH-Ag, type I) and ME49 (ME49-Ag, Type II), and 12 recombinant antigens (Rec-Ag) of *T. gondii*. The prevalence of anti-*T. gondii* IgG in the study population obtained with crude antigens was 12.2% and 11.3%, respectively (Table 2). We have previously investigated *T. gondii* prevalence in clinical healthy individuals with crude antigens of the RH strain *T. gondii*, the results obtained in this study was consistent with our previous study [20]. However, among the 173 samples which were positive, only 20 samples were positive by both RH-Ag and ME49-Ag (Table 2 and Figure 1), suggesting strong strain-specific immune-response in the population. Though the data reflected the exposure of *T. gondii* in the investigated population, the actual seroprevalence of infection may be under represented due to the fact that both RH-Ag and ME49-Ag were generated from tachyzoites. It is well known that *T. gondii* remains as the relatively quiescent bradyzoite stage in immuocompetent hosts, and comparative studies indicated that bradyzoites and tachyzoites do have different gene expression profiles, which may result in differences in elicitation of host immune responses [25]. However, due to technical difficulties in obtaining enough bradyzoites, the currently diagnostic antigens have all been obtained from the fast growing tachyzoites.

**Table 2:** **Positive serum number and prevalence of anti-*T. gondii *IgG detected with different antigens**

**Strain of *T. gondii***	**Antigen names**	**Stage and location**	**Number of samples (n = 880) with anti-*T. gondii *IgG and prevalence (%)**		
			**Independent positive rate**	**Cross-positive rate**	**Combined positive rate**
				**Inter-strains**	
RH	RH-Ag	Tachyzoite crude antigens	107 (12.2%)		
ME49	ME49-Ag	Tachyzoite crude antigens	99 (11.3%)		
	SAG5D	Tachyzoite, surface	87 (9.9%)	17.5%	
	SAG5A	Tachyzoite surface	93 (10.6%)		
RH-rAg	MIC6	Microneme	96 (10.9%)		
	SRS4	Surface	98 (11.2%)		
	GRA5	Dense granule	91 (10.4%)	15.6%	
	SAG1	Tachyzoite surface	93 (10.6%)		
ME49-rAg	SUS1	Surface	93 (10.6%)		28.2%
	SAG3	Surface	96 (10. 9%)		
	BAG1	Bradyzoite, cytoplasmic	131 (14.9%)		
RH/ME49-rAg	BSR4	Bradyzoite, surface	94 (10.7%)	19.2%	
	SRS8	Surface	104 (11.8%)		
	SRS9	Bradyzoite, surface	97 (11.0%)		

To test the differences in seroprevalence with tachyzoite- and bradyzoite-derived antigens, twelve recombinant antigens including three tachyzoite-specific, three bradyzoite-specific and six antigens likely expressed by both tachyzoites and bradyzoites (Table 2). These antigens have been previously proved to be immunogenic and frequently recognized by human antibodies [26-28]. The seroprevalence of specific IgG to the individual recombinant antigens were around 10%, with one exception, the positive rate (14.9%) of BAG1-GST was significantly higher than that observed with other recombinant antigens or even crude antigens (*p* < 0.05) (Table 2). Thus immuno-recognition of BAG1 is likely more prominent than other bradyzoite antigens during chronic infection. BAG1 was a 30 kDa cytosolic protein which is only expressed in the bradyzoites [29,30]. Immunological studies suggested that BAG1 was very immunogenic and could induce early humoral and cell-mediated immune responses upon infection in humans [31]. Thus it is not so surprising to observe a most prominent recognition of BAG1 by the sera compared to other tachyzoite- and bradyzoite-derived antigens.

On further analysis of the serum samples which were positive in the ELISA test, surprisingly it was found that the samples did not all overlapped or consistently react with the three kinds of antigen (Figure 1A). Among the 394 positive samples, 107 sera were positive with RH-Ag, 99 were positive with ME49-Ag and 248 were positive with the recombinant antigens. 20 samples which accounted for only 5.1% of all positive samples were detected by both RH-Ag and ME49-Ag. 20 samples, 5.1% of all positive sera, were detected by both RH-Ag and the Rec-Ags, and 26 sera, 6.6% of all positive samples, were detected by both ME49-Ag and the Rec-Ags (Figure 1A). Only 6 samples were positively detected in reactions with all three kinds of antigen and accounted for 1.5% of all positive samples. The reason that rAgs were more sensitive than native antigens may be due to the low concentration of the immunogenic components in the crude antigens. During *T. gondii* infection, a broad range of parasite antigens will be presented to the host immune system, thus the detection sensitivity using crude antigens will be affected by factors such as antibody affinity, antigen variation, inter-antigen interaction, and strain-specific responses. Similar results have been reported in previous studies [32,33].

Analysis of the ELISA results in the assays with recombinant antigens of RH and ME49 strains showed, however, more overlapping between different strains (Figure 1B and Table 2). Among the 248 positive samples detected with the 12 recombinant antigens of either RH-, ME49- or common (RH/ME49) type, 71 samples were positive in reactions with all three types of antigen, which accounted for 28.6% of all positive samples, even though the detection rates with strain-specific rAgs were higher. The data further supported the finding that a combination of several immune-dominant antigens will generate a higher diagnostic efficiency [32]. In contrast, 6.9% of the positive sera samples were detected by both RH-rAg and ME49-rAg, 11.3% were detected by both RH-rAg and common RH/ME49-rAg, and 9.7% were detected by both ME49-rAg and RH/ME49-rAg.

**Figure 2:**
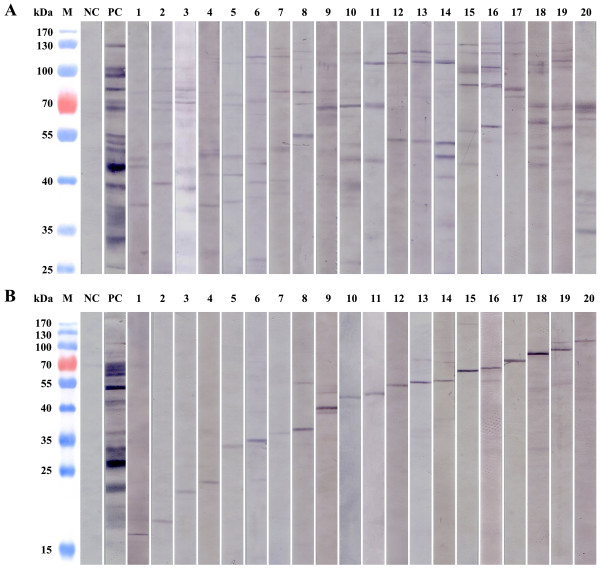
**Western blot analysis of serum samples. A** shows the examples where the serum samples recognized multiple bands of *T. gondii* proteins. **B** shows the example of the serum samples that recognized a dominant *T. gondii* protein. NC: negative control; PC: positive control; M: pre-stained Protein Molecular Weight Marker.

To determine the reliability of the ELISA results based on recombinant antigens, the 284 positive sera were further validated with two commercial *Toxoplasma gondii* IgG/IgM Kits and Western blot assays. 85.1% and 43.5% of the positive samples previously identified by the ELISA assay were confirmed by the com-IgG and com-IgM kit, respectively, whereas a 2% and 0% positive rate were found in the selected negative serum samples (*P* < 0.01) (Table 3). The data suggested, in one aspect, that half of the individuals may be recently infected by *T. gondii* at the sample time. In another aspect, a combination of antigens with different parasite strains will be needed to achieve a higher diagnostic efficiency and a broader coverage. Western blot assays showed that 71.7% and 81.5% of the ELISA positive samples were found to be immunoreactive with the native proteins of RH and ME49 strain, respectively (Table 3), which further supported the ELISA data. Examples of Western blots were shown in Figure 2 with variation in recognition of multiple bands (Figure 2A) and single bands (Figure 2B) by the serum samples. These results, again, suggested that the immune recognition of *T. gondii* antigens varied among different individuals.

**Table 3:** **The positive rates of samples detected with the commercial *T. gondii *IgG/IgM Kits and Western blot**

	**Number of positive sera by ELISA**				**Number of negative sera by ELISA**				***P *value**
	**(n = 248)**				**(n = 50, randomly selected)**				
	**positive**		**negative**		**positive**		**negative**		**(Among serum samples)**
	**n**	**%**	**n**	**%**	**n**	**%**	**n**	**%**	
com-IgG Kit	211	85.1	37	14.9	1	2	49	98	*P* < 0.01
com-IgM Kit	108	43.5	140	56.5	0	0	50	100	
WB-RH	178	71.7	70	28.3	1	2	49	98	
WB-ME49	202	81.5	46	18.5	5	10	45	90	*P* < 0.05

## Conclusions

In this study, we compared the immunorecognition of three kinds of *T. gondii* strain- and developmental status-specific antigens with the same set of sera. Results clearly showed that there were strikingly differences in the recognition of these antigens among the samples. The prevalence of anti-*T. gondii* IgG obtained with crude native RH-Ag and ME49-Ag was similar, whereas the prevalence obtained with recombinant antigens (rAgs) was significantly higher than that of the crude antigens. More importantly, the number of sera that cross-reacted with the three kinds of antigen was low, accounting for only 1.5% of the positive samples. Nevertheless the recombinant antigens had shown a significantly higher consistency in detection of *T. gondii*-specific IgG in the serum samples. Thus, the general prevalence of anti-*T. gondii* IgG was likely much higher than previously reported. The data further supported the conclusion that there is more genetic diversity among the *T. gondii* isolates in China [14-16], which argue for the necessity of the establishment of a method that can detect most, if not all, of the variant specific antibodies.

## Competing interests

The authors declare that they have no competing interests.

## Authors’ contributions

NJ, QC and XS conceived and designed the study, and critically revised the manuscript. XS, HL, BJ, ZC, SP performed the experiments, analyzed the data and drafted the manuscript. JY helped in the study design. All authors read and approved the final manuscript.
